# Sonothrombolysis Using Microfluidically Produced Microbubbles in a Murine Model of Deep Vein Thrombosis

**DOI:** 10.1007/s10439-024-03609-7

**Published:** 2024-09-09

**Authors:** Yanjun Xie, Yi Huang, Hugo C. S. Stevenson, Li Yin, Kaijie Zhang, Zain Husain Islam, William Aaron Marcum, Campbell Johnston, Nicholas Hoyt, Eric William Kent, Bowen Wang, John A. Hossack

**Affiliations:** 1 Department of Biomedical Engineering, University of Virginia, 415 Lane Road, Charlottesville, VA 22908 USA; 2 Department of Surgery, School of Medicine, University of Virginia, 409 Lane Rd MR4, Charlottesville, VA 22908 USA; 3 Feinberg School of Medicine, Northwestern University, 300 E. Superior St. Tarry Building, Chicago, IL 60611 USA

**Keywords:** Ultrasound, Deep vein thrombosis, Microfluidics, Sonothrombolysis, Microbubble

## Abstract

The need for safe and effective methods to manage deep vein thrombosis (DVT), given the risks associated with anticoagulants and thrombolytic agents, motivated research into innovative approaches to resolve blood clots. In response to this challenge, sonothrombolysis is being explored as a technique that combines microbubbles, ultrasound, and thrombolytic agents to facilitate the aggressive dissolution of thrombi. Prior studies have indicated that relatively large microbubbles accelerate the dissolution process, either in an *in vitro* or an arterial model. However, sonothrombolysis using large microbubbles must be evaluated in venous thromboembolism diseases, where blood flow velocity is not comparable. In this study, the efficacy of sonothrombolysis was validated in a murine model of pre-existing DVT. During therapy, microfluidically produced microbubbles of 18 μm diameter and recombinant tissue plasminogen activator (rt-PA) were administered through a tail vein catheter for 30 min, while ultrasound was applied to the abdominal region of the mice. Three-dimensional ultrasound scans were performed before and after therapy for quantification. The residual volume of the thrombi was 20% in animals post sonothrombolysis versus 52% without therapy ($p = 0.012 < 0.05$), indicating a significant reduction in DVT volume. Histological analysis of tissue sections confirmed a reduction in DVT volume post-therapy. Therefore, large microbubbles generated from a microfluidic device show promise in ultrasound-assisted therapy to address concerns related to venous thromboembolism.

**Supplementary Information:**

The online version contains supplementary material available at 10.1007/s10439-024-03609-7.

## Introduction

It is estimated that there are more than one million cases involving venous thromboembolism (VTE) annually in the United States. These cases include deep vein thrombosis (DVT) and pulmonary embolism (PE) [1]. DVT not only contributes to high healthcare expenses [2], but also remains responsible for a one-year mortality rate of 20% [3]. Furthermore, the occurrence of DVT and its sequelae can cause considerable disability and impaired quality of life in individuals after experiencing DVT [4, 5]. Successful treatment of DVT has been shown to be vital in improving long-term outcomes in patients [6, 7].

Standard therapies for acute VTE include oral anticoagulants and reperfusion [8]. With the wide use of oral anticoagulants in clinics, a common side effect is hemorrhage in patients with VTE [9–11]. Meanwhile, systemic infusion of thrombolytic medications, such as recombinant tissue plasminogen activator (rt-PA), results in major bleeding in approximately 3.5% of the patients [12, 13]. It is now more widely accepted that catheter-directed thrombolysis (CDT) delivers smaller doses of thrombolytic agents than systemic infusion for rapid thrombus dissolution [14, 15]. However, the large-scale randomized ATTRACT trial did not show evidence of improvements in pharmacomechanical CDT (PCDT; CDT with the use of mechanical thrombectomy devices) over oral anticoagulation alone in 2-year post-thrombotic syndrome (PTS) occurrence (47% PCDT vs 48% No-PCDT, $p=0.56$) [16, 17]. PCDT/CDT should be reserved for highly selected patients with severe symptoms and low bleeding risk. In light of the observations in CDT, there is a need for a safer and more efficient catheterization technique to achieve complete or partial resolution of venous thromboembolism without the risk of hemorrhage.

The use of ultrasound has been demonstrated to enhance the delivery of thrombolytic medications and contrast agents, such as microbubbles (MBs), into blood clots, through acoustic radiation force [18, 19], microstreaming [20, 21], and cavitation [22–24]. In this regard, ultrasound-facilitated thrombolysis, known as sonothrombolysis, has been explored extensively to accelerate blood clot dissolution [25]. The safety profile of such ultrasound-assisted approaches, including intravascular catheter-based ones, has been widely documented [26]. Multiple studies have documented the potential of sonothrombolytic therapies in the in vitro, in vivo, and ex vivo [26–29]. Unfortunately, recent clinical trials have yielded mixed results. While ultrasound-assisted therapies could similarly dissolve DVT, the available evidence did not show significant advantages over CDT therapies [30]. A meta-analysis of clinical trials found that the commercial scheme of Ultrasound-Accelerated Catheter-Directed Thrombolysis (UACDT) without MBs may not be the preferred option due to its prolonged intervention time and marginal improvement in terms of lysis rate [30]. Another clinical study demonstrated that combining therapeutic ultrasound with microbubbles reduced the duration of thrombolysis and allowed for a lower dose of thrombolytic medications [31]. Microbubbles exposed to ultrasound can improve thrombolytic outcomes and should be considered for future therapeutic applications. Studies in animals have also shown potential with the use of microbubbles designed for sonothrombolysis [29, 32, 33]. Collectively, the suboptimal efficacy of existing therapies testifies to the need for further research efforts in improving and refining sonothrombolysis.

Recently, our laboratory has reported sonothrombolysis using large microfluidically produced MBs (>15 μm diameter) [34, 35]. In the in vivo study, a 3.3-fold reduction in rt-PA dose was allowed in this stroke model. These studies used a low duty factor (< 10%), focusing solely on the mechanical effects of ultrasound, which decreased the thermal effect. Larger microbubbles, compared to commercially available ones, can carry higher momentum under acoustic radiation force [18, 19], and their cavitation can generate more energy [20, 21, 36, 37]. This increase may facilitate the deeper penetration of thrombolytic agents into the thrombus, thereby resulting in improved lysis performance in our current design than conventional microbubbles. To mitigate the risk of gas embolism, the microbubbles were formed by a nitrogen gas core encapsulated with an albumin shell, which allowed them to dissolve within one minute [35]. Their higher inner pressure compared to the environment also prevented the agglomeration of microbubbles [38, 39].

In this context, there is still an unmet need to validate the efficacy and safety in sonothrombolysis using large MBs through the use of *in vivo* models of DVT, as the mechanical properties and aging of thrombus could vary significantly from prior studies [40, 41]. During thrombosis formation, blood flow velocities differ greatly between veins and arteries. DVT is associated with slow venous flows, whereas arterial thrombosis is often caused by rupture of atherosclerotic plaques [42, 43]. Due to rapid dissolution, sonothrombolysis with these MBs does not produce bio-effects beyond the therapeutic site [35]. Meanwhile, improved efficacy allows DVT therapy with a lower dose of rt-PA than the clinical standard. Therefore, this technique can be used as an adjunctive therapy for patients who might have side effects of hemorrhage in response to thrombolytic agents.

In this work, we investigated the therapeutic efficacy of sonothrombolysis using large microfluidically generated MBs in a mouse model of pre-existing DVT. It was the first in vivo study to evaluate the influence of ultrasound and microfluidically produced MBs on venous thromboembolism. For this objective, MBs from a flow-focusing microfluidic device was generated in situ and administered to animals through venous catheterization. Microfluidically produced MBs dissolved rapidly in blood flow, thus resulting in limited off-target bioeffects. Changes in blood clot volume were monitored by 3D ultrasound imaging. Based on existing research on CDT in DVT therapy, sonothrombolysis using large microfluidically produced MBs was hypothesized to effectively dissolve the venous thrombus when administered with a reduced dose of rt-PA.

## Methods

### Fabrication of Microbubbles via Microfluidics

The mold for the microfluidic device was fabricated using the photolithography method, as described in previous research [44, 45]. The device was poured into the SU-8 mold with polydimethylsiloxane (PDMS, Sylgard 184, Dow Corning) and assembled with a clean 500-μm-thick glass wafer. The microfluidic channel had a height of 20 μm and a nozzle width of approximately 7 μm.

MBs were produced using a gas phase of 99.995% $\hbox {N}_2$ (Linde Gas, Richmond, VA, USA) and a liquid phase of bovine serum albumin (4% w/v), dextrose (10% w/v) in 0.9% saline [45]. The flow rate of the liquid phase was 20 μL/min supplied by a syringe pump (PhD 2000, Harvard Apparatus, Holliston, MA, USA), and the setting pressure of the gas phase was fixed at 84.8 kPa using a gas regulator (PC-series, Alicat Scientific, Tucson, AZ, USA). The inlets and the outlet of the microfluidic device were connected through 30-gauge inner diameter PTFE tubings (Cole-Parmer, Vernon Hills, IL, USA). The operating parameters were verified to produce MBs in the microfluidic device under a high-speed camera (SIMD24, Specialised Imaging, Tring, UK), optimized as the existing literature [34]. The microbubbles were expected to shrink and quickly dissolve into the environment [39]. Figure S4 illustrates the decrease of microbubble diameter from the production site to the microfluidic device’s outlet.

### Ultrasound Imaging

All animal experiments followed the guidelines outlined in the Guide for the Care and Use of Laboratory Animals [46] and were conducted in accordance with the approved study protocols by the Animal Care & Use Committee (ACUC) at the University of Virginia (Protocol 4327-09-23). As described in previous literature [47], a high-frequency ultrasound imaging system, Vevo 2100 (FUJIFILM VisualSonics, Toronto, ON, Canada), equipped with a linear array probe MS-550D (40 MHz center frequency) was used to acquire data. The animals were placed on a 37 °C temperature control platform and anesthetized with 1.5% v/v isoflurane. The abdomen of the mice was shaved and treated with ultrasound gel to improve imaging quality. The transducer mounted on a 3D motor module (VisualSonics, Toronto, ON, Canada) captured long-axis slices of mice at a step size of 0.03 mm using B-mode and color Doppler mode. The acquisition was also repeated for the short-axis slice at a step size of 0.1 mm. The mice showing strong color Doppler signals in the IVC were healthy, and they were not enrolled in the study. If color Doppler signals were not continuous or detected in the IVC, another imaging acquisition was performed for additional review. The 3D data collected were saved for further image analysis.

The saved 3D data were exported to 3D Slicer software [48] for annotation and segmentation. After manually labeling multiple 2D slices for a blood clot and background at each view, a grow-from-seeds algorithm was run to semi-automatically segment the blood clot [49]. The volume of the blood clot was then calculated as an output metric. The evaluation of intra- and inter-observer variability is provided in Fig. S2.

### Deliverability Study of Microfluidically Produced Microbubbles in Mice

A preliminary investigation was conducted to determine the deliverability of microfluidically produced MBs from a venous catheter to the IVC. Healthy male C57BL/6 mice (8–12 weeks old) were anesthetized with 1.5% isoflurane and placed on a heated 37 °C platform. Microfluidically produced MBs were administered to the tail vein through a catheter for 30 s. B-mode videos were recorded using the ultrasound imaging system described above at 0, 100, and 360 s after administration.

Contrast was used to describe the signal enhancement by the microbubbles. The contrast was defined as [50]

1 $$\begin{aligned} C[\text {dB}] = 10\log _{10}\frac{\mu _{v}}{\mu _b}, \end{aligned}$$

where $\mu _v$, $\mu _b$ are the mean signal powers of the regions in the IVC and the background tissue, respectively.

### Mouse Model of DVT

A murine IVC partial ligation model was utilized to mimic the venous stenosis/insufficiency and induce the development of DVT, as previously described [51–53]. In total, forty 8-to-12-week old male C57BL/6 mice (The Jackson Laboratory, Bar Harbor, ME, USA) were anesthetized and underwent a midline laparotomy on Day 0. A 30-gauge needle was placed above the IVC as the spacer, and a suture was used to ligate the IVC immediately below the venal vein. The ligation allowed for a 90% closure of the IVC lumen, which resulted in thrombosis without endothelium denudation. After removal of the needle/spacer and wound closure, the mice were recovered and monitored for postoperative care.

**Fig. 1:**
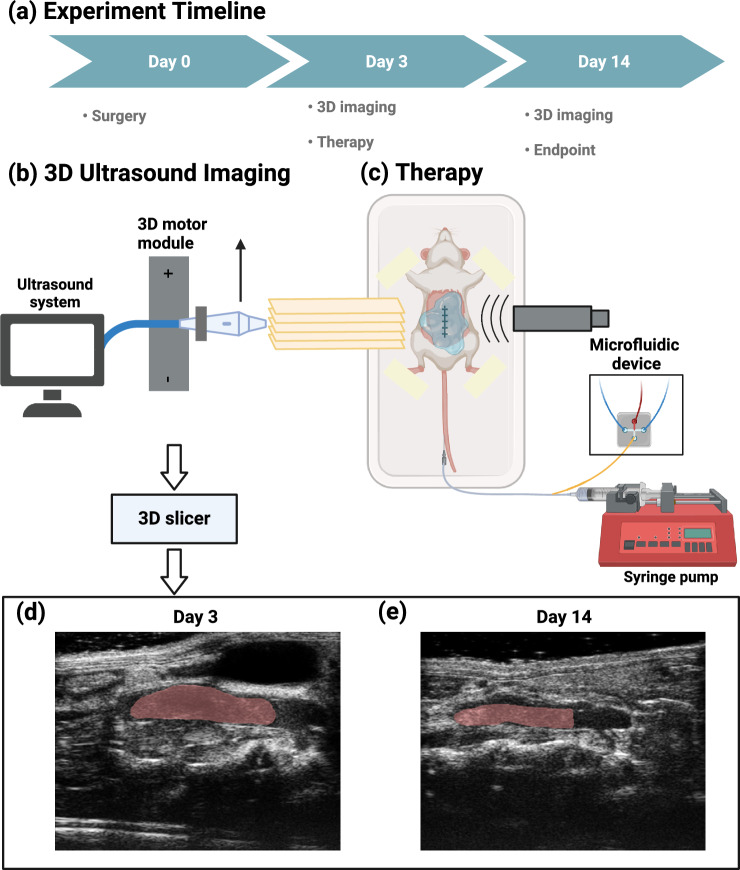
**a** Experiment timeline of this study: the animals underwent surgery of partial ligation to develop thrombus on Day 0; 3D ultrasound imaging was performed on Day 3, followed by a therapy; Another 3D ultrasound imaging was conducted on Day 14. **b** Two-dimensional slices of both long axis and short axis view of the IVC were acquired at different vessel cross-sections. Data were exported to 3D Slicer software for segmentation. **c** A therapy to mouse model of DVT. Microfluidically produced MBs and thrombolytic drugs were administered through a tail vein catheter, while ultrasound was applied from the top of the mouse abdomen. Comparison of DVT in the IVC was shown in **d** and **e**, on Days 3 and 14, respectively. The red region indicated the location of thrombus (Color figure online)

**Fig. 2:**
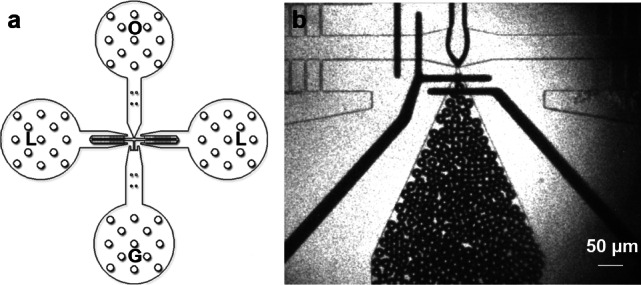
**a** Layout of a flow-focusing microfluidic device. *G* gas inlet, *L* liquid inlet, *O* MB outlet. **b** A photo of generated MBs under a high speed camera. The mean diameter of MBs was 18.0 μm

The animals were randomly divided into four different therapeutic treatments upon confirmation of DVT development on Day 3 post IVC stenosis, an intermediate time point suggested by [54]. Table 1 summarizes the treatments performed in this study. After 3D ultrasound scanning and reconstruction of the thrombi volume, animals were subjected to the aforementioned therapy session lasting 30 min. Animals in groups A, B, and C were catheterized by an experienced technician and received an injection of 2 mg/kg rt-PA (Activase, Genentech, South San Francisco, CA, USA) [55], while group D was a no-therapy control. The rt-PA dose was administered for a period of 30 min, with an initial bolus of 10%. This dose can lower the risk of major bleeding and was approximately 20% equivalent of a clinical standard dose, since mice have a 10-fold less response to human rt-PA [56, 57]. In groups A and B, a 1-MHz center frequency Panametrics ultrasound transducer (A303S, Olympus Panametrics, Waltham, MA, USA) was placed on the abdomen of mice coupled with ultrasound gel and continuously transmitted energy at a pulse repetition frequency (PRF) of 1 kHz and a duty factor of 2% during the therapy session. The low duty factor allowed for the perfusion of MBs and avoided thermal effects. The transducer was calibrated in a water tank and observed to produce a peak-negative pressure of 574 kPa, at a distance of 7 mm from the transducer surface. The corresponding intensity $I_{spta}$ was 214 mW/$\hbox {cm}^2$, determined by $I_{spta} = P_{-}^2/2\rho c\times DF$, where $P_{-}$ is the spatial-peak negative pressure, $\rho = 1000$ kg/$\hbox {m}^3$ is the density, $c = 1540$ m/s is the speed of sound and DF is the duty factor of 2% [58]. Since this intensity was below the safety limit of 720 mW/$\hbox {cm}^2$, thermal effects were negligible during the therapy using ultrasound [59, 60]. US parameters remained consistent with the calibration. Group A received microfluidically produced MBs for 30 s, every 5 min, as illustrated in Fig. 1. Image sequences of 24 frames were captured using the SIMD24 high-speed camera during the production of microbubbles [45]. In total, 20 MBs were characterized for their outer diameters. The average diameter of the MBs was measured as $18.0 \pm 1.1$ μm (mean ± SD), while the production rate was estimated to be $90.3 \times 10^3$ MBs per second. Therefore, the total gas volume was approximately 50 μL (Fig. 2).

**Table 1:** Summary of experimental groups

Group	N	rt-PA	Ultrasound	Microbubbles
A	4	+	+	+
B	4	+	+	−
C	4	+	−	−
D	4	−	−	−

On Day 14, a second imaging was performed for the animals under anesthesia, followed by euthanasia. IVC were harvested and preserved in formalin for histological purposes. The residual volume (RV) is defined as:

2 $$\begin{aligned} \text {RV}(\%) = V_{{\text{Day}} 14} /V_{{\text{Day}} 3} \times 100\%, \end{aligned}$$

where $V_{{\text{Day}}3}$, $V_{{\text{Day}}14}$ are the DVT volume segmented from acquired 3D ultrasound data on Days 3 and 14, respectively. The absolute volume change is calculated by

3 $$\begin{aligned} \varDelta V = V_{{\text{Day}} 14} - V_{{\text{Day}} 3}. \end{aligned}$$

Suggested by a prior study, the resolution of DVT can last for days even with a 10 mg/kg dose of rt-PA [54]. The period of blood clot formation in this study was analogous to that in our research. Therefore, the endpoint of this study was extended to Day 14 to measure noticeable differences in blood clot volume [61, 62].

### Histology

Excised IVC specimens were formalin-fixed and paraffin embedded. Sections of 5 μm were obtained at an interval of 0.1 mm across the samples and stained with hematoxylin and eosin (H&E) stain. Slices were scanned with a Leica microscopic camera (DMi8, Leica Microsystems, Durham, NC, USA). Representative photos of each group were selected and demonstrated.

### Statistical Analysis

Statistical analysis was performed using RStudio software (R version 4.2.1). Significance in means among all groups was determined using one-way ANOVA, followed by post hoc Fisher’s LSD test [63, 64]. The significance level was set at 0.05.

## Results

### Ultrasound Imaging Assessment of Microfluidically Produced Microbubbles in Mice

**Fig. 3:**
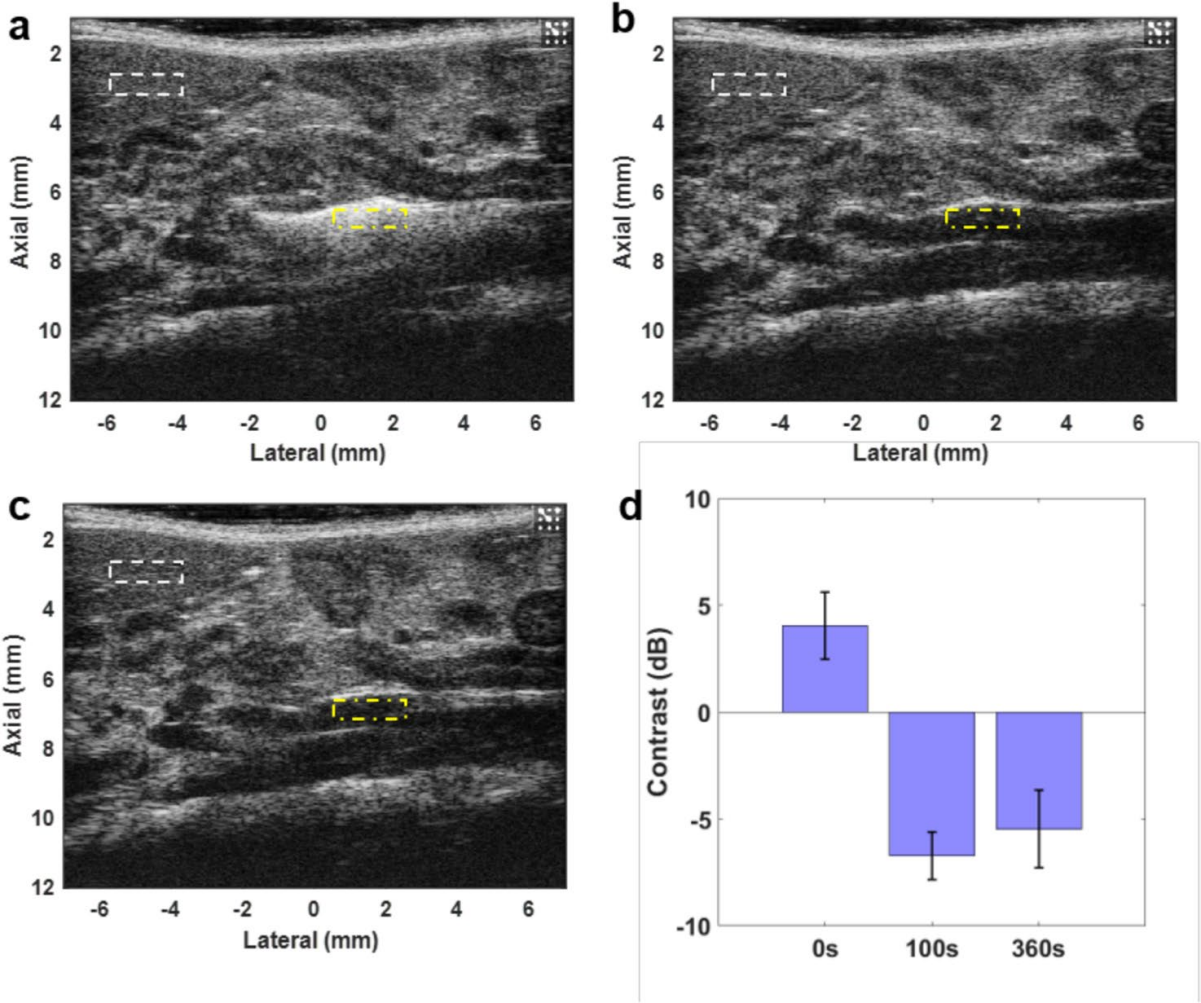
US images of IVC at **a** 0 s, **b** 100 s, and **c** 360 s after administration of microfluidically produced MBs into the catheter. Cluster of MB flow in the IVC in **a** enhanced the image intensity inside the vein. **b** A single MB traversed in the IVC. **c** No MBs were detected after 360 s. The dash-dotted and dashed rectangles in (**a**–**c**) indicate the regions of vessel and background tissue, respectively, for contrast calculation. **d** Bar plot for contrast at 0, 100, and 360 s. Error bars show the standard deviation of contrast within the videos

Figure 3 shows the B-mode imaging results in mouse IVC. Microfluidically produced MBs injected from a tail vein catheter enhanced the intensity of the B-mode image at 0 s. Due to circulation and gas dissolution, contrast enhancement weakened along with a reduction in the number of detectable MBs 100 s after injection. Eventually, there were no MBs passing through the IVC in 360 s, indicating total absorption of the MBs. The contrast between the vessel and the background tissue regions was 4.5, $-$7.3, and $-$6.7 dB at 0, 100, and 360 s, respectively. Most of MBs dissolved into blood flow and did not enhance ultrasound signal intensity at 100 s, as indicated in Fig. 3d. Microfluidically produced MBs can be delivered to the IVC and serve as a thrombolytic agent in sonothrombolysis.

### Mouse Model of DVT

**Fig. 4:**
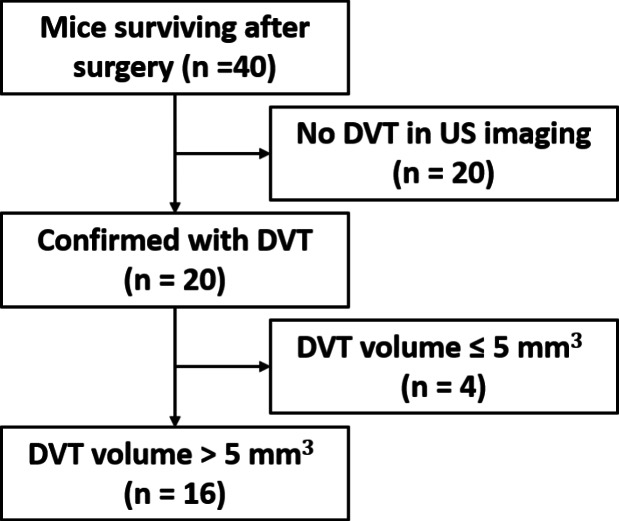
Flow diagram of mice enrollment in this study

Due to the variability inherent to murine models of DVT, not every experimental animal develops thrombosis nor uniformly. In order to focus our studies on animals with pre-existing DVT, a pre-therapy US imaging can screen those mice without DVT. The flowchart of the mice enrolled in this study is presented in Fig. 4. In total, 20 mice were confirmed with DVT by color Doppler ultrasound. Supplementary Fig. S3 displays the mice with DVT or the healthy ones. Only 16 mice with >5 $\hbox {mm}^3$ DVT were selected in the experiments. Smaller blood clots can be completely lysed without interventions and the image resolution for them may not be sufficient. All the following data were expressed as mean ± standard deviation. The mean of initial weight was $28.5 \pm 2.8$ g (A—$29.2 \pm 2.9$ g; B—$28.9 \pm 3.3$ g; C—$28.5 \pm 2.7$ g; D—$27.6 \pm 3.4$ g). The mean volume of DVT in all four groups was $15.9 \pm 4.9$ $\hbox {mm}^3$ on Day 3. The initial Day 3 volume in each group was listed below and plotted in Fig. S1: A—$20.0 \pm 6.6$ $\hbox {mm}^3$; B—$16.4 \pm 2.9$ $\hbox {mm}^3$; C—$12.5 \pm 5.1$ $\hbox {mm}^3$; D—$14.8 \pm 2.1$ $\hbox {mm}^3$. The initial volume was not significantly different ($p=0.18$

).

**Fig. 5:**
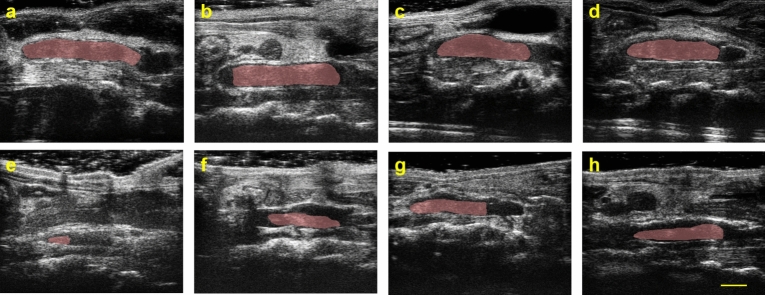
Representative ultrasound images in long-axis. The top and bottom rows are the results of Day 3 (**a**–**d**) and Day 14 (**e**–**h**), respectively. Each column is the same mouse from group A sonothrombolysis using microfluidically produced MBs (**a**, **e**), B ultrasound plus rt-PA (**b**, **f**), C rt-PA (**c**, **g**), and D no-therapy control (**d**, **h**). The red overlaid masks are the segmented blood clots. A scalebar of 2 mm is plotted

**Fig. 6:**
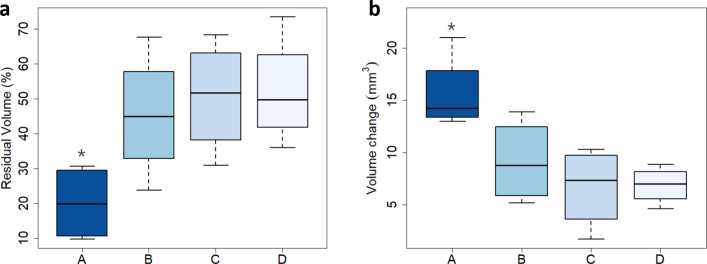
Efficacy results of therapies. **a** The residual volume of thrombus from Day 3 to Day 14 in percentage is plotted. The use of large microfluidically produced microbubbles reduces the residual volume of thrombus. The differences in the residual volume for MBs + US + rt-PA (group A) and the other groups (B: US + rt-PA; C: rt-PA and D: no-therapy control) are all statistically significant (*p* < 0.05, LSD test). **b** Absolute volume change $\varDelta V$ of each group. An asterisk denotes statistical significance of comparison to no-therapy control group D (*p* < 0.05). Additional statistical results are listed in the result section

The volume of DVT was evaluated for mice in each experimental group. Representative differences in DVT volume on Days 3 and 14 are shown in Fig. 5, and RVs are plotted in Fig. 6. The average RVs of each group were: (A) microfluidically produced MBs, ultrasound and rt-PA, $20.0 \pm 10.9 \%$; (B) ultrasound and rt-PA, $45.3 \pm 18.0 \%$; (C) rt-PA, $50.0 \pm 16.1 \%$; (D) no-therapy control, $52.2 \pm 15.7 \%$. The average absolute loss volume of each group were: (A) $15.6 \pm 3.7$ $\hbox {mm}^3$; (B) $9.2 \pm 4.0$ $\hbox {mm}^3$; (C) $6.7 \pm 3.9$ $\hbox {mm}^3$; (D) $6.9 \pm 1.8$ $\hbox {mm}^3$.

According to the ANOVA analysis, the average RV for each group was significantly different ($p = 0.0406$). The RV for group A of sonothrombolysis using MBs was significantly different from group B of ultrasound plus rt-PA ($p = 0.0387$), group C of rt-PA alone ($p = 0.0158$), and no-therapy control group D ($p = 0.0121$). Among groups B, C, and D, the RV for group B ultrasound plus rt-PA was not significantly different from group C rt-PA alone ($p = 0.636$) and no-therapy control group D ($p = 0.539$), neither between group C rt-PA alone and no-therapy control group D ($p = 0.886$).

Similarly, the absolute loss volume showed a significant difference among the four groups ($p = 0.0105$). The absolute reduction volume for group A was significantly different compared to group B ($p=0.0216$), group C ($p=0.0032$), and group D ($p=0.0037$). No significant differences were found among groups B, C, and D.

### Histology Results

**Fig. 7:**
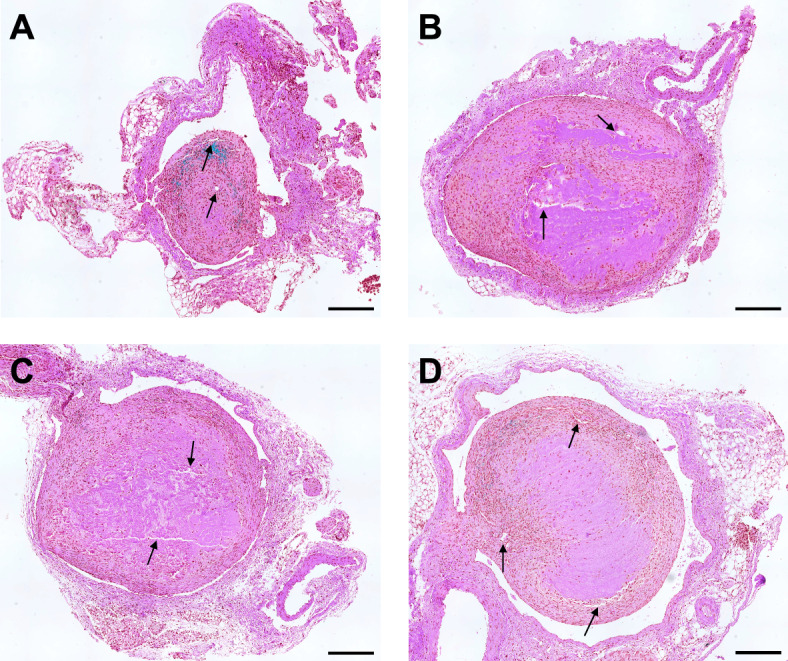
H&E staining results in **a** MBs + US + rt-PA, **b** US + rt-PA, **c** rt-PA, and **d** no-therapy control groups. Arrows in **a**–**d** show the recanalization channels in thrombus. The scale bar corresponds to 200 $\mu$m. *H* & *E* hematoxylin and eosin, *MB* microbubble, *US* ultrasound, *rt-PA* recombinant tissue plasminogen activator

Representative images of circumferential IVC samples are shown in Fig. 7. The lumen in Fig. 7a was observed with a larger space because the residual blood clot was the smallest among all sections. All residual blood clots in Fig. 7a–d were attached to the IVC vessel walls. The channels within the thrombi were found to allow recanalization, as indicated by the arrows, similar to [65–67], and the fibrin-rich region within the blood clot decreased in group A of sonothrombolysis.

## Discussion

This study was the first to evaluate the efficacy of sonothrombolysis using microfluidically produced microbubbles (>15 μm) in a murine model of DVT. Microbubbles from a microfluidic device were transiently stable, dissolved in the circulation system in minutes (Fig. 3). The use of microbubbles of large diameter in sonothrombolysis applications has previously been proven safe [35]. However, conditions (such as blood flow velocity) in the arteries and veins are not comparable. In addition to differences in arterial and venous thrombus, the age of the thrombus can also affect the therapeutic efficacy of a certain therapy. Catheter-directed intervention for DVT does not show clinical improvement over anticoagulant medications in early-stage DVT, but it is at increased risk of vein injury [17, 68–70]. An appropriate intervention time point for intravenous therapy should be selected for thrombolysis studies. Li et al. propose that patients with intermediate DVT may experience better clinical outcomes when treated with catheter-directed thrombolysis [54]. Therefore, our sonothrombolysis was performed on Day 3 post the surgery. The dissolution of DVT was a gradual process, and the differences in DVT volume required time to be measured [54]. Our findings of a 14-day timeline can contribute to future research into PTS.

Our mouse model of DVT was created by partial ligation of the IVC, leading to venous stenosis and hemodynamic instability. This model allows for DVT formation while preserving blood flow as observed in clinical DVTs, thereby enabling the delivery of thrombolytic drugs and microbubbles to blood clots. Therefore, it is a suitable animal model for thrombolysis studies. However, this model is associated with a large variance in thrombus size [71]. To mitigate differences in blood clot size, in this study, blood clots less than 5 $\hbox {mm}^3$ were excluded after pre-therapy ultrasound imaging, as in Fig. 4, since smaller clots were generally easier to remove and low risk, even without interventions. In the *in vivo* experiments, the residual volume rate in rt-PA (group C) was not significantly different from the no-therapy control group D, which was consistent with the results of rats with acute carotid artery occlusion [55]. The sub-clinical 2 mg/kg dose of rt-PA did provide insufficient lysis efficacy. The thrombus volume in the no-therapy control group D decreased by approximately 50% over a period of 14 days, which aligns with the findings of previous studies [72–74]. Furthermore, sonothrombolysis using large microbubbles (group A) displayed significantly improved therapeutic efficacy compared to other control groups in Fig. 6, matching results in the *in vitro* model and the arterial model [34, 35].

The microfluidically produced microbubbles were of 18.0 μm diameter at a production rate of approximately 90$\times 10^3$ MBs/s. Large microbubbles have been shown to have stronger bioeffects in applications of blood brain barrier disruption [37], stroke [35], and DVT [27, 34]. A possible explanation for this phenomenon is the increased transfer of momentum induced by acoustic radiation force [18, 19], and higher-energy cavitation events [20, 21]. The administration of microbubbles and thrombolytic agents was performed through a tail vein catheter. Therefore, the routes of intravenous administration should be chosen between a tail vein [35] or femoral veins [75, 76], as they were physically close to the location of the thrombus in the IVC. However, femoral vein injections presented technical difficulties and were not suitable for a study involving long-term survival of mice [77]. Given the number of animals, we use the current scheme to evaluate our performance. Notably, in our experiments, the administration of microbubbles and rt-PA did not result in any mouse fatalities.

Ultrasound applied to cavitate the microbubbles was implemented by an Olympus Panametrics transducer on the abdomen. Catheter-based ultrasound has also been investigated to enable human-compatible dimensions, such as a forward-looking transducer that generates vortex ultrasound [26, 78, 79], or a side-looking transducer [14, 29, 80, 81]. These therapeutic ultrasound designs can be combined with real-time control of the on-chip microbubbles generation [45]. Advances in the miniaturization of therapeutic ultrasound and microfluidic technology will provide effective therapies for thrombus-related conditions, in the field of catheter-directed therapy. This is an important step toward the miniaturization of human-compatible microfluidic devices for DVT therapy.

The segmentation of the DVT was mostly based on static ultrasound signals. One limitation was due to low sensitivity in the presence of thick adipose tissue. To improve detection of the blood clot boundary, we manually adjusted the imaging plane from the center of abdomen when the imaging quality was affected by shadowing of tissue. The initialization of the algorithm still relied on manual annotation of the thrombus and background [49]. A fully automatic machine learning method can reduce the bias of different experts and generate consistent results among all groups [82]. However, collection and organization of 3D ultrasound dataset of mouse models of DVT might be needed and then used to train or fine-tune a neural network [83]. Representative histological images in Fig. 7 match the quantitative results provided by ultrasound. The smallest cross-sectional area of the residual blood clot is observed in the MBs + US + rt-PA group (A) compared to the other slices. Due to the 50% successful rate of the surgery, the sample size was limited ($N=4$) and the experiments were conducted in the current conditions. Previous studies have demonstrated the reduction of rt-PA using large microbubbles [34, 35]. Therefore, this research focused on the efficacy of our sonothrombolysis technique in the DVT model. Though the absolute change in thrombus volume (Fig. 6b) was associated with the initial volume, the initial volume was not significantly different in this study. We chose to use a relative volume metric to represent efficacy.

This study faces certain limitations. First, the stenosis model was used to replicate the reopened thrombus in DVT patients. This model exhibits large variation in thrombus size and fails to mimic a complete occlusion situation. Other models of DVT, such as the stasis model or the electrolytic IVC model, will be used to examine the effectiveness of our sonothrombolysis technique. Secondly, only the symptoms of DVT or bleeding were monitored following the therapy. For clinical translation, it is worth noting that none of the commonly used murine models of DVT - IVC stenosis, IVC stasis (complete ligation), and electric injury - develop PE spontaneously. Recent studies have reported the potential utility of femoral vein stenosis combined with light illumination in recapitulating PE, although further characterization of its robustness and clinical relevance is still warranted [84]. Alternatively, an IVC filter can be implanted to avoid the formation of embolism [85]. In the future, the long-term outcomes using large microbubbles and ultrasound will be investigated. It is essential to assess its efficacy against PTS, which affects patients’ quality of life yet still without effective treatments. By leveraging micro-manufacturing techniques, the combination of ultrasound transducers with microfluidic devices will allow for a potential new approach to the resolution of thrombus. In addition to the DVT/PE application considered here, the method has a potential role in the context of cerebral vessels—i.e., stroke. A catheter could be placed within millimeters of the target usage site and generate microbubbles on-site for sonothrombolysis.

## Conclusion

At a reduced dose of rt-PA to lessen risk of hemorrhage, large microbubbles were used as a therapeutic agent in a mouse model of DVT, combining with ultrasound and rt-PA. The transiently stable microbubbles produced by a microfluidic device did not cause safety issues in this study, and their use in sonothrombolysis resulted in an improvement in the dissolution of DVT. More research is needed to evaluate the applicability of this technique to other DVT models and its long-term outcomes for PTS. This may involve the development of a human qualified prototype, followed by validation in large animals and then in humans under the appropriate safety controls.

## Supplementary Information

Below is the link to the electronic supplementary material.

Supplementary file 1 (pdf 778 KB)
