# Supplementary material for: Sonothrombolysis Using Microfluidically Produced Microbubbles in a Murine Model of Deep Vein Thrombosis
Source: Ann Biomed Eng. 2024 Sep 9;53(1):109–19. doi: 10.1007/s10439-024-03609-7 (PMC11782319; doi:10.1007/s10439-024-03609-7)
Supplement: Supplementary file 1 — (pdf 778 KB) [file 10439_2024_3609_MOESM1_ESM.pdf]

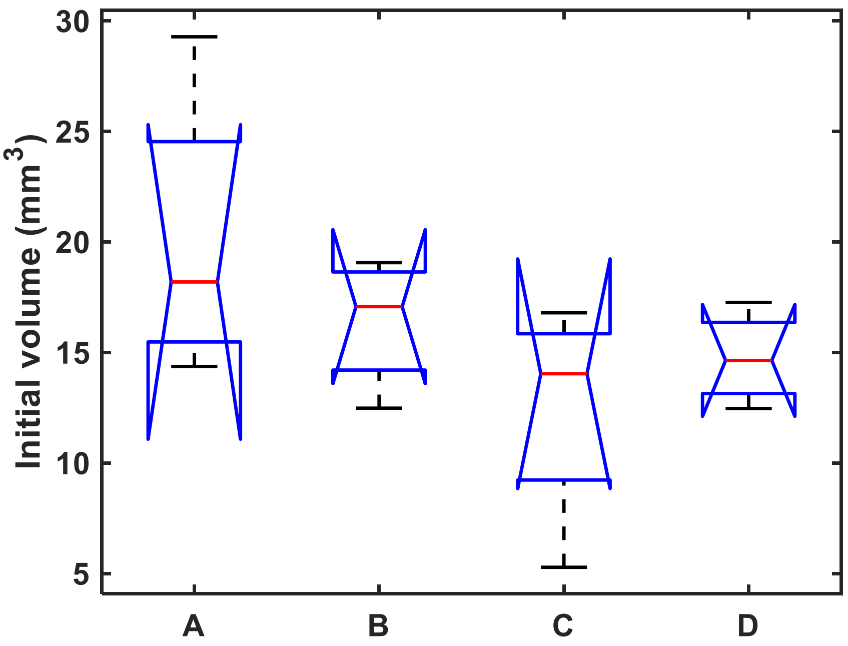

Figure S1. Boxplot of initial volume in each group. A, 20 ± 6.6 mm^3; B, 16.4 ± 2.9 mm^3; C, 12.5 ± 5.1 mm^3; D, 14.8 ± 2.1 mm^3 (mean ± SD). No significant difference is found using one-way ANOVA test ($p = 0.18$).

Two observers manually annotated blood clots and background of 7 subjects, and then ran the segmentation algorithm. The differences between the first and the second observers were calculated. For another 7 subjects, an observer measured them twice to compute the differences between the two observations.

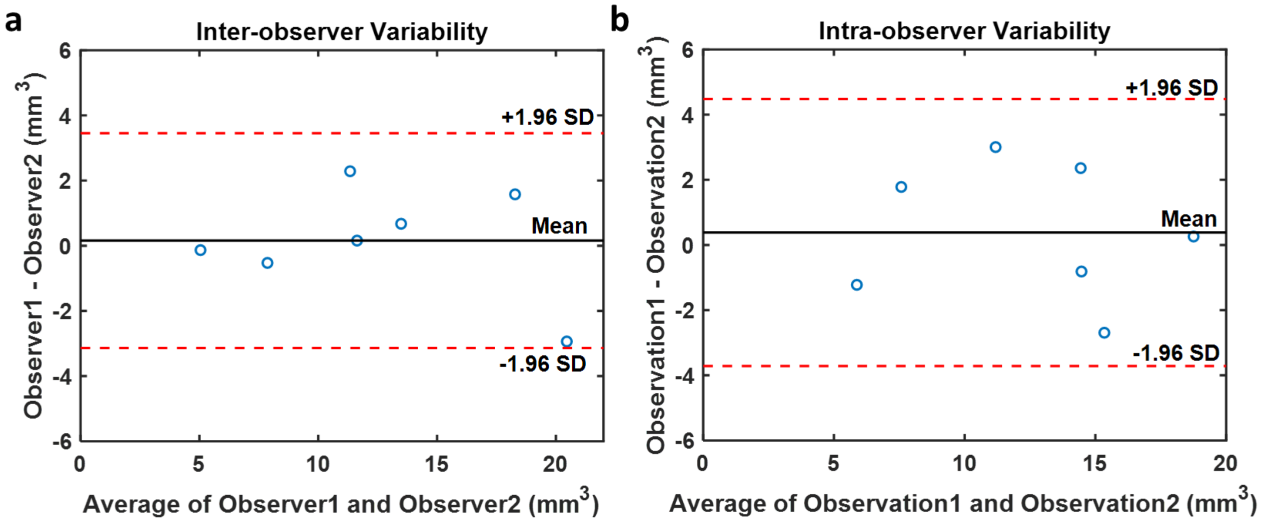

Figure S2. The inter- and intra-observer variability. (a) The mean difference between the two observers' volume in the same subject was 0.16 ± 1.68 mm^3 (mean ± SD). (b) The mean within-subject difference between the first and the second observation is 0.38 ± 2.09 mm^3 (mean ± SD).

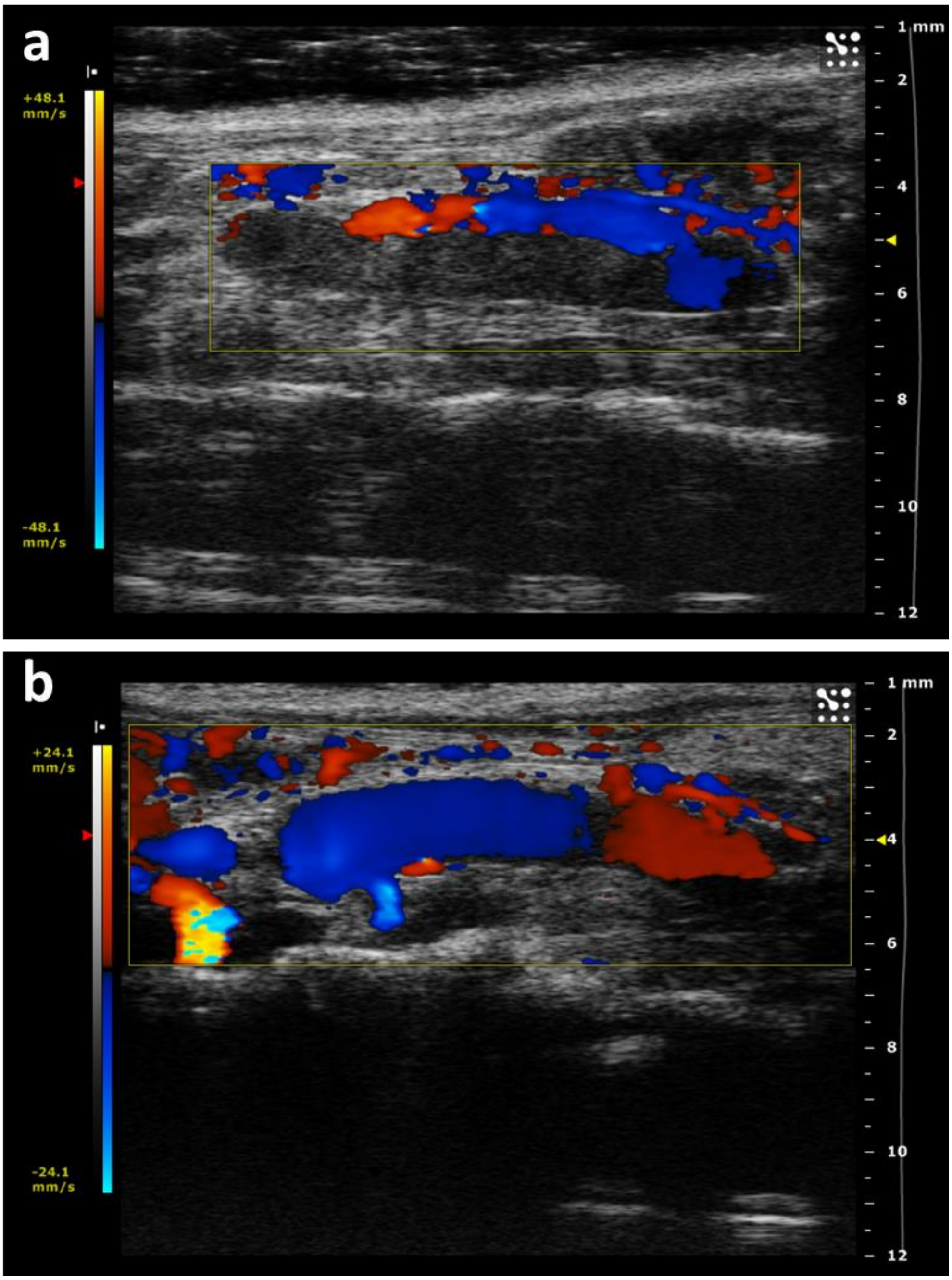

Figure S3. Example color Doppler and B-mode images of (a) the IVC with a thrombus, and (b) normal IVC, respectively. (a) The thrombus occludes the blood flow and presents static speckle in the vein. (b) The blood flow in the vein is continuous.

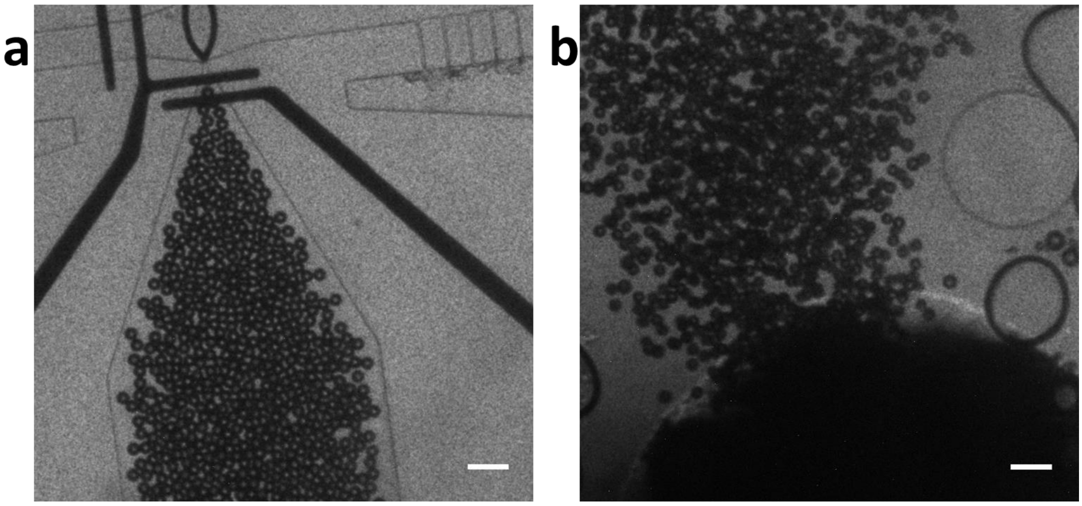

Figure S4. Photos of Microbubbles at (a) the production nozzle and (b) microfluidic device's outlet, under a high-speed camera. The average diameter is (a) 5.8 ± 0.9 µm and (b) 14.6 ± 1.3 µm, decreasing 7.6% in a 3-mm long channel.
